# Biological aging, circulating lipid biomarkers and pancreatic diseases: a prospective cohort study based on the UK Biobank

**DOI:** 10.1016/j.jnha.2026.100982

**Published:** 2026-09-15

**Authors:** Hang Zhang, Junwei Huang, Peiyu Lin, Jiayin Song, Chonghui Hu, Biaojun Lin, Lingdan Le, Shangyou Zheng

**Affiliations:** a The Third Clinical College, Guangzhou Medical University, Guangzhou, Guangdong,511436, China; b School of Medicine, Jinan University, Guangzhou, Guangdong,510630, China; c School of Health Management, Guangzhou Medical University, Guangzhou, Guangdong,511436, China; d Third Affiliated Hospital of Guangzhou Medical University, Guangzhou, Guangdong,510150, China; e Department of Pancreas Center, Department of General Surgery, Guangdong Provincial People's Hospital (Guangdong Academy of Medical Sciences), Southern Medical University, Guangzhou, Guangdong, 510080, China; f Department of Oncology, Sun Yat-sen Memorial Hospital of Sun Yat-sen University, Guangzhou, Guangdong, 510120, China

**Keywords:** Pancreatic diseases, Phenotypic age acceleration, Biological age acceleration, Circulating lipid biomarkers

## Abstract

**Background:**

Biological aging refers to gradual declines in overall physiological function and can raise susceptibility to diseases. However, whether biological age acceleration is associated with pancreatic diseases remains unclear.

**Methods:**

In UK Biobank, 326,873 adults recruited in 2006–2010 were included. Klemera-Doubal Method Biological Age Acceleration (KDMAgeAccel) and Phenotypic Age Acceleration (PhenoAgeAccel) were categorized (G1–G4 by standard deviation [SD]) and modeled continuously. We examined the associations and dose-response relationships between acceleration and the following clinical outcomes: overall pancreatic diseases, acute pancreatitis, chronic pancreatitis, pancreatic cysts, pancreatic cancer, and pancreas-related mortality. Cohen’s d, independent t-tests, multivariable Cox proportional hazards regression, and causal mediation analysis were used to assess differences between aging groups, disease associations, and the mediation effects of circulating lipid biomarkers.

**Results:**

KDMAgeAccel and PhenoAgeAccel demonstrated significant associations with pancreatic disease risk (both P < 0.05). PhenoAgeAccel demonstrated significant non-linear associations with overall pancreatic diseases, pancreatic cysts, pancreatic cancer, and pancreas-related mortality (all P for non-linearity <0.05), while no significant non-linearity was observed for acute pancreatitis or chronic pancreatitis (both P for non-linearity >0.05). In the fully adjusted Cox model, compared with G1, the PhenoAgeAccel G4 group had higher risks of all pancreatic diseases (hazard ratio [HR] 1.706, 95% confidence interval [CI] 1.513–1.923), acute pancreatitis (1.637, 1.352–1.981), chronic pancreatitis (3.551, 2.248–5.609), pancreatic cysts (1.547, 1.128–2.122), pancreatic cancer (1.387, 1.123–1.713), and pancreas-related mortality (1.442, 1.139–1.824).Circulating lipid biomarkers partially mediated the association between PhenoAgeAccel and pancreatic diseases, with notable contributions from free cholesterol in small LDL (6.66% for chronic pancreatitis), cholesterol to total lipids ratio in medium LDL (8.54% for acute pancreatitis), and cholesterol to total lipids ratio in large LDL (8.06% for pancreatic cancer).

**Conclusions:**

Aging biomarkers, particularly in accelerated aging populations, are significantly associated with pancreatic disease risk, with potential implications for clinical risk stratification. Circulating lipid biomarkers were identified as potential statistical mediators of the association between PhenoAgeAccel and pancreatic diseases.

## 1 Introduction

The pancreas is a pivotal organ with both exocrine and endocrine functions [[1], [2], [3], [4]]. In recent years, there has been a significant increase in the incidence of pancreatic diseases, including chronic pancreatitis, acute pancreatitis, and pancreatic cancer, which have become important causes of morbidity and mortality worldwide [[5], [6], [7]]. Both pancreatic diseases and diabetes mellitus increase the risk of developing pancreatic cancer. Pancreatic cancer remains one of the most lethal malignancies worldwide, characterized by late diagnosis, rapid progression, and limited treatment options. Although recent data show that survival for patients with localized disease is substantially higher (44–46%), the overall 5‑year survival rate of pancreatic cancer remains low at 12–13% [8,9]. Therefore, elucidating the risk factors and underlying mechanisms of pancreatic diseases and pancreatic cancer is of critical importance.

Aging is a progressive process of functional decline in the body at the molecular, cellular, tissue, and organ levels. It can increase disease susceptibility by inducing cell death, promoting disease progression, exacerbating inflammatory microenvironments, and disrupting tissue homeostasis [[10], [11], [12], [13]].Consequently, aging significantly elevates the risk of metabolic disorders. As a key metabolic organ, the pancreas undergoes both structural and functional alterations during the aging process, which may be closely associated with the development of pancreatic diseases. However, chronological age merely reflects the passage of time and fails to capture inter-individual variability in the rate of aging and the extent of functional decline [[14], [15], [16], [17], [18]]. To address this limitation, several biological age metrics have been developed, including the Klemera–Doubal method biological age (KDMAge) and phenotypic age (PhenoAge). Building on these measures, age acceleration indices quantify the deviation of biological age from chronological age, thereby reflecting the relative pace of aging. This provides a novel quantitative framework for investigating the relationship between aging and disease onset. Although recent studies have explored the role of biological age acceleration in cardiovascular diseases, malignancies, and neurodegenerative disorders [19,20], evidence in the context of pancreatic diseases remains limited, particularly with respect to comprehensive assessments across multiple pancreatic outcomes.

From a mechanistic perspective, aging-related physiological changes may induce systemic remodeling of lipid metabolism, including alterations in lipoprotein subfractions and fatty acid composition [21,22]. Previous studies have demonstrated that pancreatic lipid accumulation is closely associated with an increased risk of pancreatitis and pancreatic cancer [[23], [24], [25]]. Lipid metabolic dysregulation may represent a potential mechanism linking biological age acceleration to pancreatic diseases.

Based on this background, the present study leverages data from the UK Biobank, a large-scale prospective cohort, to systematically evaluate the associations of KDMAgeAccel and PhenoAgeAccel with the incidence of pancreatic diseases and related mortality. Furthermore, we aim to assess their potential utility in risk prediction and to investigate the mediating role of lipid subfractions. This study seeks to provide novel epidemiological evidence for the early identification of pancreatic diseases from the perspective of systemic physiological aging.

## 2 Methods

### 2.1 Study population

The original data of this study were derived from the UK Biobank (UKB) [26], which recruited over 500,000 participants aged 40–69 years between 2006 and 2010. Participants were assessed across 22 evaluation centers in England, Wales, and Scotland. They completed self-administered touchscreen questionnaires and underwent anthropometric measurements. Baseline assessments included detailed exposure history, health status, and collection of biological samples for multi-platform analyses. The study was approved by the Northwest Multi-Centre Research Ethics Committee, the National Information Governance Board for England and Wales, and the Community Health Index Advisory Group in Scotland.

From an initial baseline cohort of 502,157 participants, the following exclusion criteria were sequentially applied to define the final analytical sample for this prospective study: (1) Participants were excluded due to missing biological aging-related indicators. (2) Participants who withdrew their informed consent during the study period. (3) Participants whose pancreatic outcomes occurred prior to the baseline assessment. (4) Participants with a history of pancreatic transplantation or who are in a pancreatic transplantation status at baseline. (5) Participants who were lost to follow-up. (6) Participants with missing values in key covariates.

### 2.2 Calculation of KDM/Phenotypic age and KDM/Phenotypic age acceleration

Two rigorously validated biological age (BA) algorithms, Klemera-Doubal method biological age (KDMAge) and phenotypic age (PhenoAge), both based on clinical and blood biochemical markers, were used in this study. Klemera-Doubal method (KDM) age was proposed by Klemera and Doubal and derived from clinical biomarkers [[27], [28], [29], [30]], including total cholesterol, forced expiratory volume in 1 second, systolic blood pressure, albumin, blood urea nitrogen, creatinine, C-reactive protein, glycated hemoglobin, and alkaline phosphatase [[31], [32], [33], [34]]. PhenoAge was derived from 9 blood biochemical indicators: albumin, alkaline phosphatase, creatinine, C-reactive protein (CRP), glucose, mean corpuscular volume, red blood cell distribution width, white blood cell count, and lymphocyte percentage [[35], [36], [37], [38]]. The residuals from the regression of the two resulting Bioages on chronological age were recorded as Klemera-Doubal Method Biological Age Acceleration and Phenotypic Age Acceleration (KDMAgeAccel and PhenoAgeAccel), respectively [[39], [40], [41], [42], [43]]. Detailed calculation steps and explanations of the two biological age measurement methods are provided in Supplementary Material 1. For categorical analysis, participants were divided into four groups based on the standard deviation (SD) of the entire cohort's age acceleration distribution: Group 1 (< −1 SD, G1), Group 2 (−1 SD to 0, G2), Group 3 (0 to +1 SD, G3), and Group 4 (> +1 SD, G4). G1, which represented participants with the lowest degree of biological age acceleration, was defined as the reference group.

### 2.3 Outcome definition

Study outcomes were ascertained through linkage to UK National Health Service databases, including hospitalization records and national death registries. Primary endpoints comprised acute pancreatitis, chronic pancreatitis, pancreatic cysts, pancreatic cancer, a composite endpoint of all pancreatic diseases, and pancreas-related mortality. Case definitions were based on International Classification of Diseases (ICD) codes (Supplementary Table S6). Incident pancreatic events during follow-up were analyzed as time-to-event outcomes, with follow-up time calculated from baseline assessment until the occurrence of pancreatic outcomes, death, loss to follow-up, or the end of follow-up, whichever occurred first.

### 2.4 Covariates

Potential confounding factors were identified based on prior knowledge and biological plausibility. The following covariates were included in all multivariable models: age at baseline (continuous); sex (male or female); ethnicity (White or nonWhite); Townsend deprivation index (continuous, with higher values indicating greater material deprivation); educational attainment (High, Intermediate, and Low/Other); smoking status (never, previous, or current); alcohol intake frequency (Never/Other, <3 times/week, or ≥3 times/week); physical activity (meeting or not meeting the physical activity recommendation); body mass index (BMI; <25, 25–30, or >30 kg/m^2^); sleep duration (continuous, hours per day); PM₂.₅ exposure (continuous, μg/m³); diabetes mellitus (present or absent); Cholelithiasis (present or absent); and lipid-lowering therapy use (yes or no). The same covariate set was applied consistently in all multivariable Cox regression models, dose-response analyses, sensitivity analyses, and mediation analyses. For certain categorical variables, variables that could not be meaningfully classified or defined were excluded prior to analysis. For continuous variables, missing values were imputed using mean imputation.

### 2.5 Mediation analysis

To evaluate whether lipid subfractions statistically mediate the associations between PhenoAgeAccel and pancreatic disease outcomes, we performed causal mediation analyses within a counterfactual framework using the “CMAverse” R package (version 0.3.0) with the regression-based method [44]. The outcome was modeled with a Cox proportional hazards model to appropriately account for right-censored survival data, while the mediator was modeled with linear regression. We estimated the proportion mediated (PM) using the imputation-based approach and obtained 95% confidence intervals from 1,000 non-parametric bootstrap resamples. After excluding 23 non-lipid metabolites (amino acids, glycolysis-related metabolites, ketone bodies, fluid balance markers, and inflammatory glycoproteins), 228 circulating lipid biomarkers across 13 categories were retained as candidate mediators, following the UK Biobank lipid biomarker classification (Resource 3543). Before inclusion in the mediation models, mediator variables were winsorized at the 0.1 st and 99.9th percentiles to limit the influence of extreme observations. Candidate mediators were first screened by requiring an absolute Cohen's d > 0.2 with PhenoAgeAccel. Retained variables were then required to demonstrate significant associations with pancreatic outcomes in multivariable Cox models (Benjamini–Hochberg adjusted P < 0.05), followed by a third filter requiring significant indirect effects in the mediation analysis (Benjamini–Hochberg adjusted P < 0.05).

### 2.6 Statistical analysis

Kaplan-Meier analysis was used to generate cumulative risk curves stratified by KDMAgeAccel or PhenoAgeAccel, with between-group differences assessed by log-rank tests. Cox proportional hazards models were employed to estimate hazard ratios (HRs) and 95% confidence intervals (CIs) for associations between aging acceleration measures and incident pancreatic outcomes. All analyses were adjusted for the same comprehensive set of covariates described in the Methods. For each endpoint, we utilized Cox regression with restricted cubic splines (RCS) to model time-to-event data and visualize potential dose-response relationships. RCS models used the same covariate adjustment strategy as the primary Cox regression models. Associations were assessed both categorically (comparing G2-G4 against G1) and continuously across age acceleration, with separate estimates calculated for acceleration (>0) and deceleration (<0) phases. In a sensitivity analysis, participants who developed the corresponding pancreatic outcome within the first two years after baseline were excluded from that outcome-specific analysis to evaluate whether early disease onset influenced the observed associations and to reduce potential bias caused by pre-existing subclinical pancreatic disease. Additionally, we evaluated the associations between biological age acceleration and lipid mediators. Cohen’s d and independent t-tests were employed to evaluate effect size differences in lipid mediators between groups, while multivariable Cox proportional hazards regression was used to assess the associations between lipid-related variables and pancreatic disease outcomes. Each lipid mediator was standardized to Z-scores (mean = 0, SD = 1).

## 3 Results

### 3.1 Study participants

**Fig. 1:**
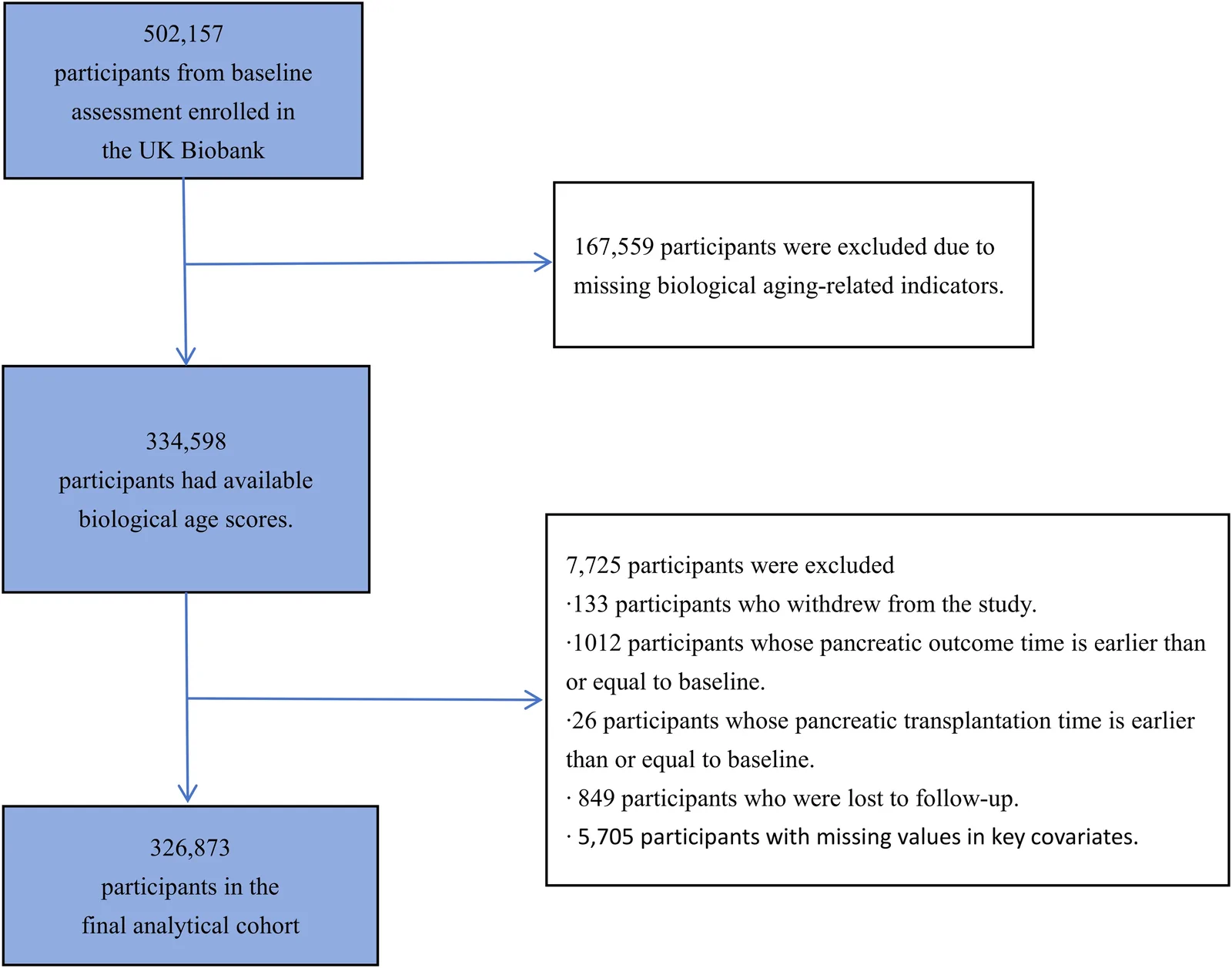
Flow diagram for the selection of the study population of the UK Biobank.

As illustrated in Fig. 1, following the sequential exclusions (including missing biological age, withdrawal, pancreatic outcome/transplantation time earlier than baseline, loss to follow-up, or missing key covariates), a total of 326,873 participants were included in the final analysis, comprising 322,456 controls without pancreatic diseases and 4,417 cases. The 4,417 cases consisted of 1,883 acute pancreatitis, 443 chronic pancreatitis, 577 pancreatic cysts, 1,375 pancreatic cancer, and 1,068 pancreas-related mortality events (some participants had multiple diagnoses). As summarized in Table 1, participants with pancreatic diseases were generally older, had a higher proportion of males, current smokers, and obesity (BMI ≥ 30), along with lower educational attainment and distinct alcohol consumption patterns. A higher prevalence of diabetes mellitus (more than twice that of controls), gallstone disease, lipid-lowering therapy use, greater material deprivation (Townsend index), slightly higher PM₂.₅ exposure, and slightly shorter sleep duration were also observed in the case group.

**Table 1:** Baseline characteristics of participants.

	Control	Pancreatic diseases	Acute pancreatitis	Chronic pancreatitis	Cyst of pancreas	Pancreatic cancer	Pancreas-related mortality
N	322456	4417	1883	443	577	1375	1068
Age (mean (SD))	56.34 (8.09)	59.70 (7.27)	58.35 (7.92)	58.71 (7.75)	59.64 (7.08)	61.17 (6.28)	61.63 (6.18)
Male (%)	147876 (45.86)	2189 (49.56)	918 (48.75)	291 (65.69)	229 (39.69)	749 (54.47)	586 (54.87)
White ethnicity (%)	293473 (91.01)	4055 (91.80)	1748 (92.83)	398 (89.84)	527 (91.33)	1248 (90.76)	980 (91.76)
Education level (%)							
Low/Other	69569 (21.57)	1346 (30.47)	574 (30.48)	151 (34.09)	162 (28.08)	420 (30.55)	350 (32.77)
Intermediate	146739 (45.51)	1903 (43.08)	852 (45.25)	189 (42.66)	260 (45.06)	539 (39.20)	413 (38.67)
High	106148 (32.92)	1168 (26.44)	457 (24.27)	103 (23.25)	155 (26.86)	416 (30.25)	305 (28.56)
Townsend deprivation index (mean (SD))	−1.40 (3.02)	−0.98 (3.23)	−0.88 (3.22)	−0.24 (3.51)	−1.05 (3.21)	−1.22 (3.19)	−1.27 (3.15)
Smoking status (%)							
Never smoker	178373 (55.32)	2092 (47.36)	903 (47.96)	162 (36.57)	297 (51.47)	642 (46.69)	491 (45.97)
Previous smoker	111466 (34.57)	1725 (39.05)	744 (39.51)	167 (37.70)	223 (38.65)	543 (39.49)	421 (39.42)
Current smoker	32617 (10.12)	600 (13.58)	236 (12.53)	114 (25.73)	57 (9.88)	190 (13.82)	156 (14.61)
Alcohol intake frequency (%)							
Never/Other	24229 (7.51)	433 (9.80)	198 (10.52)	60 (13.54)	51 (8.84)	97 (7.05)	83 (7.77)
<3times/week	155560 (48.24)	2171 (49.15)	988 (52.47)	182 (41.08)	302 (52.34)	615 (44.73)	479 (44.85)
≥3times/week	142667 (44.24)	1813 (41.05)	697 (37.02)	201 (45.37)	224 (38.82)	663 (48.22)	506 (47.38)
Physical activity (%)	115450 (35.80)	1768 (40.03)	780 (41.42)	199 (44.92)	227 (39.34)	510 (37.09)	405 (37.92)
BMI category (%)							
<25 kg/m^2^	108601 (33.68)	1078 (24.41)	376 (19.97)	133 (30.02)	189 (32.76)	345 (25.09)	260 (24.34)
25–30 kg/m^2^	137975 (42.79)	1854 (41.97)	765 (40.63)	181 (40.86)	242 (41.94)	611 (44.44)	473 (44.29)
>30 kg/m^2^	75880 (23.53)	1485 (33.62)	742 (39.41)	129 (29.12)	146 (25.30)	419 (30.47)	335 (31.37)
Sleep duration (mean (SD))	7.11 (1.23)	7.06 (1.42)	7.01 (1.50)	6.86 (1.71)	7.05 (1.31)	7.13 (1.27)	7.18 (1.28)
PM₂.₅(mean (SD))	9.97 (1.03)	10.04 (1.04)	10.06 (1.00)	10.14 (1.08)	10.06 (1.07)	9.96 (1.03)	9.97 (1.03)
Diabetes mellitus (%)	4942 (1.53)	173 (3.92)	57 (3.03)	38 (8.58)	22 (3.81)	44 (3.20)	35 (3.28)
Cholelithiasis (%)	5133 (1.59)	146 (3.31)	69 (3.66)	19 (4.29)	25 (4.33)	25 (1.82)	15 (1.40)
Lipid-lowering therapy (%)	55483 (17.21)	1071 (24.25)	432 (22.94)	132 (29.80)	136 (23.57)	336 (24.44)	279 (26.12)
KDMAgeAccel (mean (SD))	−0.04 (4.51)	0.64 (4.77)	0.83 (4.66)	1.13 (5.20)	0.25 (4.93)	0.25 (4.60)	0.25 (4.66)
PhenoAgeAccel (mean (SD))	−0.05 (5.35)	1.42 (6.52)	1.32 (6.19)	3.46 (7.23)	0.87 (6.38)	1.00 (6.20)	1.17 (6.35)

Abbreviations: BMI, body mass index; PM₂.₅, particulate matter with aerodynamic diameter ≤2.5 μm; KDMAgeAccel, Klemera-Doubal Method Biological Age Acceleration; PhenoAgeAccel, Phenotypic Age Acceleration.

The analysis included 49,293, 120,003, 108,978, and 48,599 participants in KDMAgeAccel groups G1 to G4, and 39,221, 144,416, 104,250, and 38,986 in the corresponding PhenoAgeAccel groups. The pancreatic disease group exhibited significantly higher mean KDMAgeAccel (0.64 ± 4.77 vs. −0.04 ± 4.51) and PhenoAgeAccel (1.42 ± 6.52 vs. −0.05 ± 5.35) compared to controls.

### 3.2 Cumulative risk and dose–response of aging with pancreatic outcomes

**Fig. 2:**
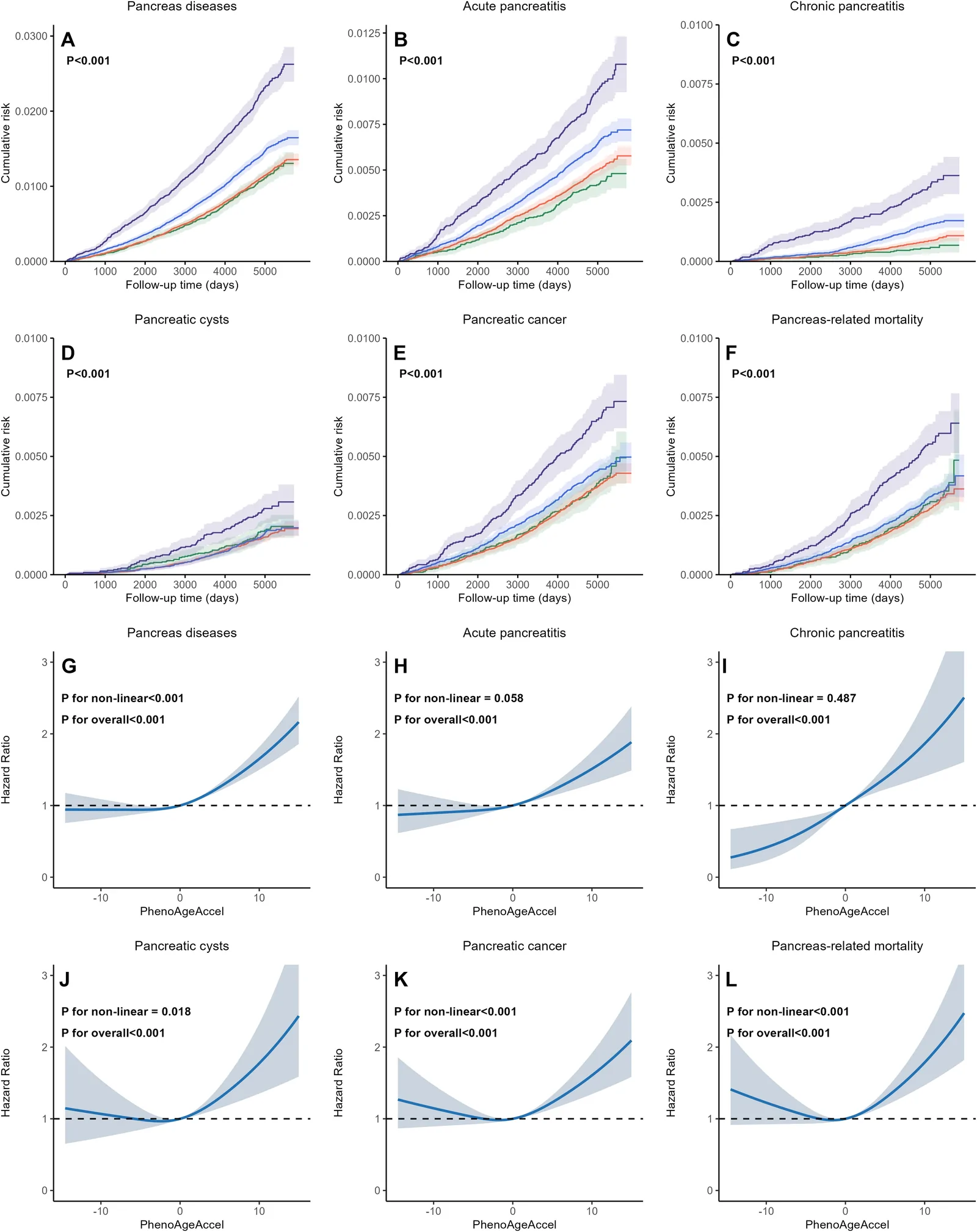
Associations and Dose-Response Relationships of PhenoAgeAccel with Pancreatic Disease Risk. (A–F) Kaplan–Meier curves showing cumulative risk of overall pancreatic diseases (A), acute pancreatitis (B), chronic pancreatitis (C), pancreatic cysts (D), pancreatic cancer (E), and pancreas-related mortality (F) across PhenoAgeAccel groups. Higher levels of PhenoAgeAccel were associated with progressively increased cumulative risk (log-rank P < 0.001 for all comparisons). (G–L) Restricted cubic spline analyses based on Cox proportional hazards models illustrating the dose–response relationships between continuous PhenoAgeAccel and each outcome. Solid lines represent hazard ratios, and shaded areas indicate 95% confidence intervals. All Cox regression models were adjusted for age, sex, ethnicity, smoking status, alcohol intake, physical activity, body mass index, sleep duration, educational attainment, Townsend deprivation index, PM₂.₅ exposure, diabetes mellitus, Cholelithiasis, and lipid-lowering therapy use.

Kaplan–Meier analysis based on the two aging acceleration measures showed that PhenoAgeAccel was associated with significant differences in all six endpoints: pancreatic diseases, acute pancreatitis, chronic pancreatitis, pancreatic cysts, pancreatic cancer, and pancreas-related mortality (all P < 0.001) (Fig. 2A–F).In contrast, KDMAgeAccel showed significant differences in pancreatic diseases, acute pancreatitis, and chronic pancreatitis (all P < 0.001), but not in pancreatic cysts (P = 0.452), pancreatic cancer (P = 0.059), or pancreas-related mortality (P = 0.127) (Figure S1A–F).Regarding the dose-response relationship between KDMAgeAccel/PhenoAgeAccel and pancreatic disease risk, KDMAgeAccel showed significant associations only with the overall risk of pancreatic diseases, acute pancreatitis, and chronic pancreatitis (all P for overall <0.05). These associations were predominantly linear, with evidence of non-linearity limited to chronic pancreatitis (P for non-linearity = 0.004) (Figure S1G–L); in contrast, PhenoAgeAccel was associated with all pancreatic disease endpoints (all P for overall <0.001) and demonstrated significant non-linear relationships for overall pancreatic diseases, pancreatic cysts, pancreatic cancer, and pancreas-related mortality (all P for non-linearity <0.05), but not for acute pancreatitis (P = 0.058) or chronic pancreatitis (P = 0.487) (Fig. 2G–L).

### 3.3 Pancreatic disease risk in multivariate cox models

**Fig. 3:**
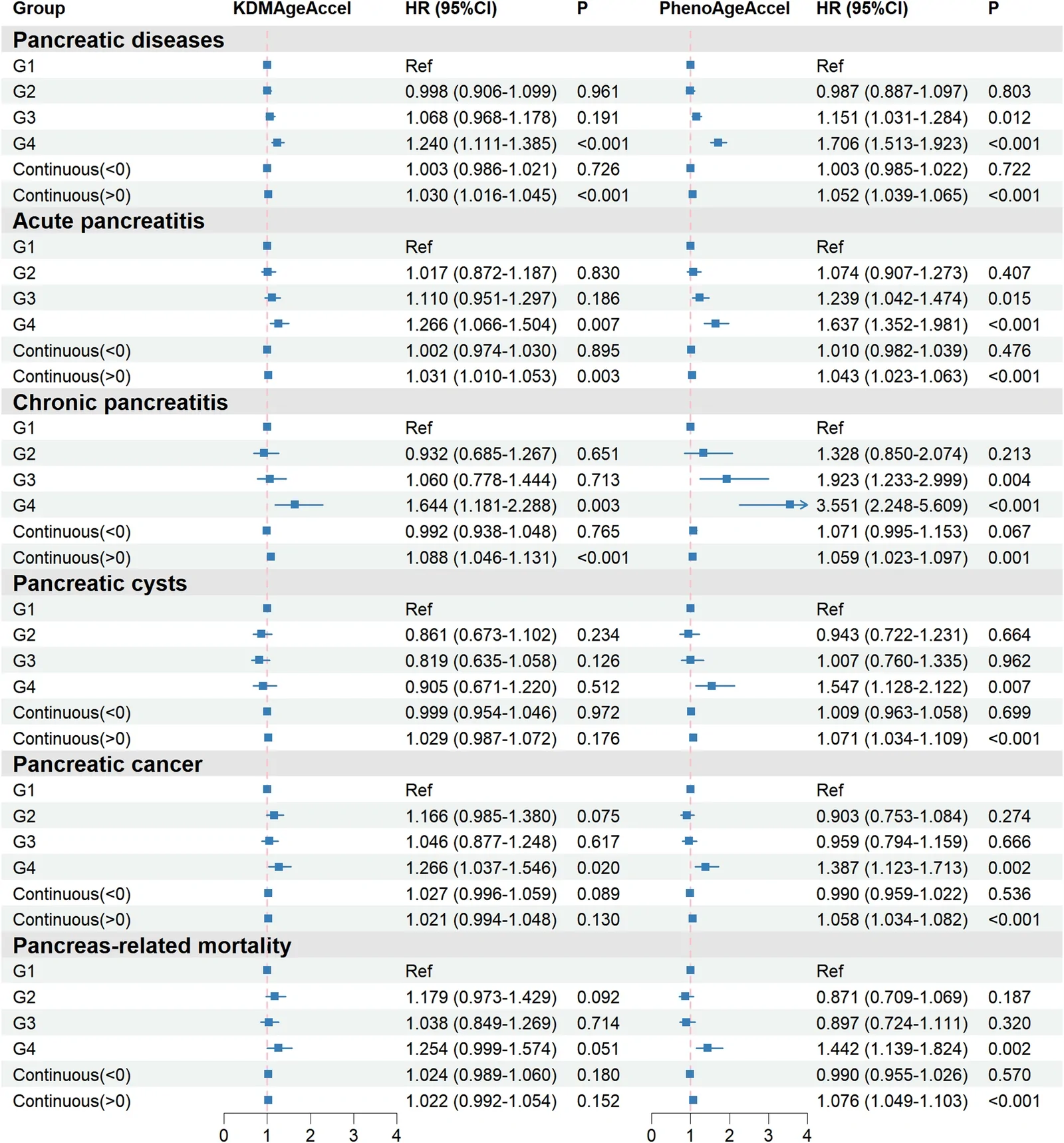
Risk of Pancreatic Diseases Based on KDMAgeAccel and PhenoAgeAccel. "Ref" (reference) denotes the reference group (Group G1).After adjusting for chronological age, sex, ethnicity, Townsend deprivation index, educational attainment, smoking status, alcohol intake frequency, physical activity, body mass index, sleep duration, PM₂.₅ exposure, diabetes mellitus, cholelithiasis, and lipid-lowering therapy use, hazard ratios (HRs) for pancreatic-related outcomes (Pancreatic Diseases, Acute Pancreatitis, Chronic Pancreatitis, Pancreatic Cysts, Pancreatic Cancer, and pancreas-related mortality) across different groups were estimated using Cox regression models

After adjusting for confounders, the multivariate Cox model (Fig. 3) revealed that both KDMAgeAccel and PhenoAgeAccel were independently associated with pancreatic disease outcomes, though the strength and extent of associations differed. Using G1 as reference, KDMAgeAccel G4 was associated with significantly increased risks of overall pancreatic diseases (HR = 1.240, 95% CI 1.111–1.385, P < 0.001), acute pancreatitis (HR = 1.266, 95% CI 1.066–1.504, P = 0.007), and chronic pancreatitis (HR = 1.644, 95% CI 1.181–2.288, P = 0.003), as well as pancreatic cancer (HR = 1.266, 95% CI 1.037–1.546, P = 0.020), but not pancreatic cysts (HR = 0.905, 95% CI 0.671–1.220, P = 0.512). In continuous analysis, each 1‑unit increase in KDMAgeAccel during the accelerated aging phase (>0) was linked to elevated risks of overall pancreatic diseases, acute pancreatitis, and chronic pancreatitis, whereas no significant associations were observed in the decelerated phase (<0). In contrast, PhenoAgeAccel demonstrated stronger and more pervasive associations. Participants in the PhenoAgeAccel G4 group showed significantly elevated risks for overall pancreatic diseases, acute pancreatitis, chronic pancreatitis, pancreatic cysts, pancreatic cancer, and pancreas‑related mortality. In the fully adjusted model, PhenoAgeAccel G4 vs G1 hazard ratios were: all pancreatic diseases (HR = 1.706, 95% CI 1.513–1.923, P < 0.001); acute pancreatitis (HR = 1.637, 95% CI 1.352–1.981, P < 0.001); chronic pancreatitis (HR = 3.551, 95% CI 2.248–5.609, P < 0.001); pancreatic cysts (HR = 1.547, 95% CI 1.128–2.122, P = 0.007); pancreatic cancer (HR = 1.387, 95% CI 1.123–1.713, P = 0.002); and pancreas‑related mortality (HR = 1.442, 95% CI 1.139–1.824, P = 0.002). When analyzed continuously, each 1‑unit increase in PhenoAgeAccel (acceleration phase) was associated with a significant increase in risk across all pancreatic outcomes, whereas no significant associations were observed in the decelerated phase.

### 3.4 Mediation analysis

**Fig. 4:**
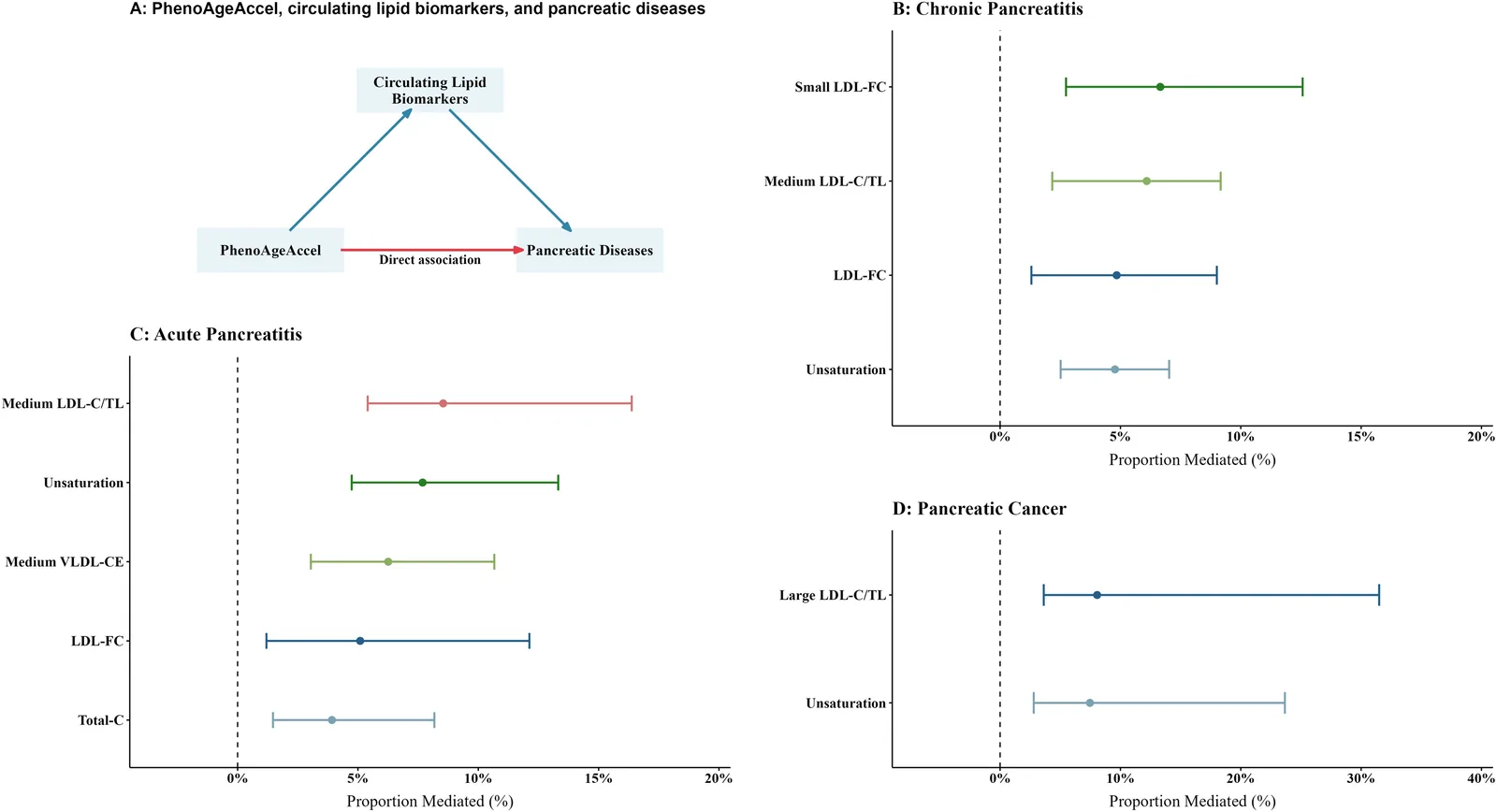
Mediation of the PhenoAgeAccel–Pancreatic Diseases Association by Circulating Lipid Biomarkers. (A) Conceptual framework illustrating direct and indirect statistical associations linking PhenoAgeAccel to pancreatic diseases through circulating lipid biomarkers. (B–D) Proportion mediated (%) by individual lipid subfractions for chronic pancreatitis (B), acute pancreatitis (C), and pancreatic cancer (D). Only lipid biomarkers with both significant mediation effects and the largest mediated proportion within each lipid subgroup for each outcome are shown. All models were adjusted for chronological age, sex, ethnicity, Townsend deprivation index, educational attainment, smoking status, alcohol intake frequency, physical activity, body mass index, sleep duration, PM₂.₅ exposure, diabetes mellitus, cholelithiasis, and lipid-lowering therapy use; error bars represent 95% CI.

Abbreviations: C, cholesterol; CE, cholesteryl esters; DHA, docosahexaenoic acid; FC, free cholesterol; HDL, high-density lipoprotein; IDL, intermediate-density lipoprotein; LDL, low-density lipoprotein; MUFA, monounsaturated fatty acids; PL, phospholipids; PUFA, polyunsaturated fatty acids; SM, sphingomyelins; TG, triglycerides; TL, total lipids; VLDL, very low-density lipoprotein.

We started with 228 circulating lipid biomarkers and applied a three‑step selection procedure. First, variables with an absolute Cohen's d > 0.2 with PhenoAgeAccel were retained, yielding 46 unique metabolites. Second, 38 metabolites remained after Benjamini–Hochberg correction (adjusted P < 0.05) in multivariable Cox models (38 for acute pancreatitis, 22 for chronic pancreatitis, and 14 for pancreatic cancer). Third, following the same correction for indirect effects in mediation analysis, 32 metabolites remained significant overall (29 for acute pancreatitis, 19 for chronic pancreatitis, and 6 for pancreatic cancer), with some metabolites overlapping across outcomes. (Detailed mediation estimates are provided in Supplementary Tables S1–S3, and the metabolites with the largest mediated proportions in each outcome are displayed in Fig. 4). In chronic pancreatitis, free cholesterol in small LDL had the highest mediated proportion (6.66%; 95% CI 2.74%–12.57%), followed by cholesterol to total lipids ratio in medium LDL (6.09%; 95% CI 2.17%–9.16%), free cholesterol in LDL (4.85%; 95% CI 1.30%–9.00%), and degree of unsaturation (4.77%; 95% CI 2.52%–7.02%). In acute pancreatitis, the largest mediator was cholesterol to total lipids ratio in medium LDL (8.54%; 95% CI 5.40%–16.37%), followed by degree of unsaturation (7.69%; 95% CI 4.74%–13.32%), cholesteryl esters in medium very low-density lipoprotein (VLDL) (6.26%; 95% CI 3.04%–10.66%), free cholesterol in LDL (5.09%; 95% CI 1.20%–12.12%), and total cholesterol (3.92%; 95% CI 1.47%–8.18%). For pancreatic cancer, the strongest mediator was the cholesterol to total lipids ratio in large LDL (8.06%; 95% CI 3.63%–31.50%), followed by degree of unsaturation (7.47%; 95% CI 2.79%–23.66%).

### 3.5 Sensitivity analysis

**Fig. 5:**
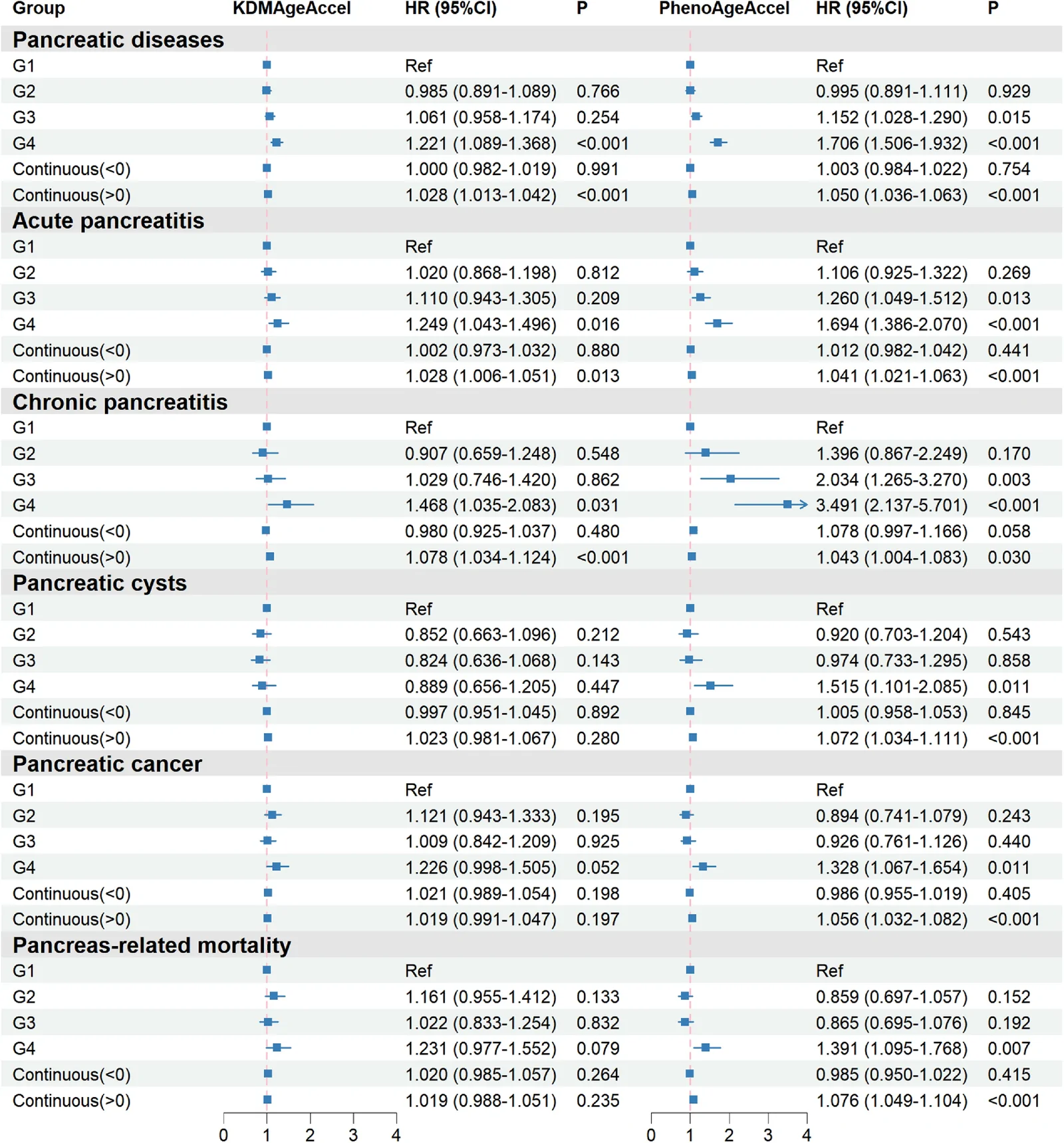
Sensitivity Analysis: Adjusted Hazard Ratios for Pancreatic Outcomes by KDMAgeAccel and PhenoAgeAccel. Associations of KDMAgeAccel and PhenoAgeAccel with pancreatic outcomes were estimated using multivariable Cox proportional hazards models. This sensitivity analysis excludes participants who developed the corresponding pancreatic outcome within the first two years of follow-up from that outcome-specific analysis, to mitigate potential reverse causality. Results are shown for categorical exposure (SD-based groups G1–G4, with G1 as reference) and continuous exposure (stratified by accelerated aging (>0) and decelerated aging (<0) phases). KDMAgeAccel and PhenoAgeAccel exhibit differential associations with overall pancreatic diseases, acute pancreatitis, chronic pancreatitis, pancreatic cysts, pancreatic cancer, and pancreas-related mortality, with PhenoAgeAccel showing more pervasive significant risks across outcomes. All models were adjusted for chronological age, sex, ethnicity, Townsend deprivation index, educational attainment, smoking status, alcohol intake frequency, physical activity, body mass index, sleep duration, PM₂.₅ exposure, diabetes mellitus, cholelithiasis, and lipid-lowering therapy use; error bars represent 95% CI.

In the sensitivity analysis (Fig. 5), for PhenoAgeAccel, significant associations were observed with multiple pancreatic outcomes in the highest SD-based category (G4): overall pancreatic diseases (HR = 1.706, 95% CI 1.506–1.932, P < 0.001), acute pancreatitis (HR = 1.694, 95% CI 1.386–2.070, P < 0.001), chronic pancreatitis (HR = 3.491, 95% CI 2.137–5.701, P < 0.001), pancreatic cysts (HR = 1.515, 95% CI 1.101–2.085, P = 0.011), pancreatic cancer (HR = 1.328, 95% CI 1.067–1.654, P = 0.011), and pancreas-related mortality (HR = 1.391, 95% CI 1.095–1.768, P = 0.007). In contrast, KDMAgeAccel was significantly associated with overall pancreatic diseases (G4: HR = 1.221, 95% CI 1.089–1.368, P < 0.001), acute pancreatitis (G4: HR = 1.249, 95% CI 1.043–1.496, P = 0.016), and chronic pancreatitis (G4: HR = 1.468, 95% CI 1.035–2.083, P = 0.031). Associations with other outcomes—pancreatic cysts, pancreatic cancer, and pancreas-related mortality—were not significant.

## 4 Discussion

Based on data from the UK Biobank cohort, this study systematically evaluated the associations of two biological aging acceleration indicators—KDMAgeAccel and PhenoAgeAccel—with the risk of pancreatic diseases and pancreas-related mortality. Both aging acceleration indicators were independently associated with pancreatic disease risk, with PhenoAgeAccel showing consistently stronger associations, particularly with chronic pancreatitis. Circulating lipid biomarkers partially mediated the PhenoAgeAccel–pancreatic disease associations, suggesting that lipid-related metabolic changes may contribute to aging-related pancreatic pathology.

Biological age acceleration reflects cumulative declines in physiological function and has been associated with a range of age-related conditions, including cardiovascular diseases, malignancies, and neurodegenerative disorders [19,20]. It may help identify individuals at elevated risk of pancreatic diseases, potentially complementing conventional approaches that rely on imaging abnormalities or overt clinical symptoms. Between the two measures, PhenoAgeAccel showed consistently stronger and more pervasive associations than KDMAgeAccel across all pancreatic outcomes. This difference likely stems from the inclusion of multiple hematological indicators in the PhenoAge model—mean corpuscular volume, red blood cell distribution width, white blood cell count, and lymphocyte percentage—which together place greater weight on immune and inflammatory pathways compared with the KDM model [45]. PhenoAgeAccel also exhibited non-linear associations with several outcomes, characterized by a more pronounced increase in risk at higher levels of PhenoAgeAccel. The aging metrics examined here are derived from routinely collected clinical and biochemical data (e.g., complete blood counts and basic metabolic parameters), offering advantages of accessibility, low cost, and non-invasiveness [46,47]. These characteristics suggest that these biomarkers may have potential utility for risk stratification in primary care and community settings; however, their screening performance, clinical utility, and preventive value require formal evaluation in dedicated future studies. Both indicators showed the strongest associations in the highest SD-based category (G4).

Although the pathological hallmarks of these conditions differ—acute pancreatitis involves acute inflammatory injury, chronic pancreatitis features progressive fibrosis, pancreatic cysts carry variable malignant potential, and pancreatic cancer arises from genetic alterations within a complex tumor microenvironment—dysregulated lipid and lipoprotein metabolism has been implicated across all of them [[48], [49], [50]].In acute and chronic pancreatitis, altered lipid profiles and the accumulation of certain lipoprotein subclasses may contribute to inflammatory injury and disease progression [51].In pancreatic cancer, lipid metabolic reprogramming, including changes in cholesterol and fatty acid metabolism, is increasingly recognized as a hallmark of the tumor microenvironment [52,53]. Notably, biological aging is itself accompanied by progressive alterations in lipid metabolism, such as shifts in lipoprotein subclass distribution and fatty acid composition [54]. These shared metabolic features may, at least in part, explain why aging-related lipid metabolic changes were linked to multiple pancreatic outcomes in our study.

Mediation analysis revealed that circulating lipid biomarkers partially mediated the association between PhenoAgeAccel and pancreatic diseases, with mediation proportions ranging from 0.59% to 8.54%. Although modest, this mediating effect was consistently observed and biologically plausible. The relatively low mediation proportion may be attributable to several factors: measurement of individual metabolites may not fully capture systemic lipid metabolic status; single-timepoint assessments cannot reflect dynamic metabolic changes; and distinct lipid subfractions may contribute differentially to specific pancreatic disease outcomes, suggesting the presence of highly selective specific metabolic pathways. These findings imply that aging-related metabolic dysregulation does not operate through global lipid abnormalities, but rather through specific lipid pathways that mediate pancreatic pathology. Future studies are needed to identify the specific lipid subfractions that mediate aging-related pancreatic damage and to evaluate whether modulating these subfractions can attenuate such damage. Such work would also help clarify how a common aging process can drive pathological changes in different organs.

Our study has several notable strengths. These include the use of a large and well-characterized cohort with long-term follow-up, the comprehensive assessment of multiple pancreatic disease outcomes, and the application of validated biological aging biomarkers. In addition, supplementary analyses in accelerated aging subgroups and sensitivity analyses excluding early events yielded consistent results, further supporting the robustness of the main findings. Nevertheless, several limitations should also be acknowledged. The observational design of this study limits causal inference, and residual confounding may still exist despite extensive adjustment for potential covariates. Although participants who were lost to follow-up were excluded, the exclusion of individuals with missing data may have introduced selection bias, and the potential for survivor bias cannot be fully excluded. Furthermore, the use of a single population-based cohort may limit the generalizability of our findings, and external validation in independent cohorts is required. Finally, our analyses provide limited mechanistic insight into the biological pathways linking accelerated aging with pancreatic diseases. Prospective studies in diverse populations, together with mechanistic investigations, are needed to clarify the biological pathways linking accelerated aging with pancreatic diseases.

In summary, the present findings suggest that aging biomarkers, particularly PhenoAgeAccel, are associated with pancreatic disease risk, with stronger associations in high-risk subgroups and consistent results across sensitivity analyses. These findings highlight the potential utility of aging biomarkers for identifying individuals at higher risk, and formal evaluation of their predictive performance and clinical utility warrants further investigation in future clinical and translational studies. Additionally, circulating lipid biomarkers were identified as potential statistical mediators of the association between aging acceleration and pancreatic diseases, offering epidemiological insights into the possible biological pathways underlying these associations.

## Author contributions

Hang Zhang: Data curation; formal analysis; methodology; software; writing – original draft.

Junwei Huang: Data curation; formal analysis; software; writing – original draft.

Peiyu Lin: Data curation; formal analysis; software; writing – original draft.

Jiayin Song: Data curation; formal analysis; software; writing – original draft.

Chonghui Hu: funding acquisition; writing – review & editing.

Biaojun Lin: Conceptualization; methodology; project administration; supervision.

Lingdan Le: writing – review & editing; supervision.

Shangyou Zheng: funding acquisition; writing – review & editing.

## Ethical approval

All procedures performed in studies involving human participants were in accordance with the ethical standards of the institutional and/or national research committee and with the 1964 Helsinki declaration and its later amendments or comparable ethical standards.

## Animal rights

Not applicable. The UK Biobank study received ethical approval from the North West Multi-Centre Research Ethics Committee (REC reference: 11/NW/0382). This research was conducted under UK Biobank application number 85224.

## Declaration of Generative AI and AI-assisted technologies in the writing process

During the preparation of this manuscript, the authors used generative AI tools only for language polishing and improving readability. All scientific content, study design, data analysis, interpretation of results, and conclusions were independently developed and verified by the authors. No generative AI or AI-assisted technologies were used to generate data, perform statistical analyses, or create figures, images, and artwork.

## Funding

This study was supported by grants from the 10.13039/501100001809National Natural Science Foundation of China (grant numbers 82372859 and 82573586 to S.Z., 82072639 and 82203691 to C.H.), 10.13039/501100003453Natural Science Foundation of Guangdong Province (grant number 2024A1515013216 to C.H.), the Guangzhou Science and Technology Bureau Basic and Applied Research Project (grant numbers 2025A04J4765 to S.Z.).

## Data availability statement

Data used in this study are from the UK Biobank and are available to bona fide researchers through an application process described at https://biobank.ctsu.ox.ac.uk/.

## Declaration of competing interest

The authors declare that they have no known competing financial interests or personal relationships that could have appeared to influence the work reported in this paper.
