# Supplementary material for: Biological aging, circulating lipid biomarkers and pancreatic diseases: a prospective cohort study based on the UK Biobank
Source: J Nutr Health Aging. 2026 Sep 15;30(11):100982. doi: 10.1016/j.jnha.2026.100982 (PMC13594620; doi:10.1016/j.jnha.2026.100982)
Supplement: Supplementary file 1 [file mmc2.docx]

**Supplementary materials**

***Supplementary Table S5 Data field and description of basic information in UK Biobank***

| Basic information | Data field | Description |
| --- | --- | --- |
| Age | 21022 | Age at recruitment |
| Sex | 31 | Sex at recruitment |
| Townsend | 22189 | Townsend deprivation index at recruitment |
| Ethnic | 21000 | Ethnic background |
| BMI | 21001 | Body mass index (BMI) |
| Alcohol intake frequency | 1558 | Alcohol intake frequency |
| Smoking | 20116 | Smoking status |
| Qualification | 6138 | Category of qualification |
| Sleep duration | 1160 | Sleep duration in every 24 hours |
| Physical activity | 22036 | At or above moderate/vigorous/walking recommendation |
| PM2.5 | 24006 | Particulate matter air pollution (pm2.5) |
| Treatment/medication code | 20003 | Self-reported regular medication use |
| Date of death | 40000 | Date of death |
| Date of cancer diagnosis | 40005 | National cancer registry |
| Date of first in-patient diagnosis - ICD10 | 41280 | Hospital inpatient |
| Date of first in-patient diagnosis - ICD9 | 41281 | Hospital inpatient |
| Cause of death | 40001 | Underlying (primary) cause of death: ICD10 |
| Circulating lipid biomarkers | 23400–23648  (excluding 23460–23480) | 228 lipid biomarkers across 13 categories: cholesterol, triglycerides, phospholipids, cholesteryl esters, free cholesterol, total lipids, lipoprotein particle concentrations, lipoprotein particle sizes, other lipids, apolipoproteins, fatty acids, relative lipoprotein lipid concentrations, and lipoprotein subclasses (Supplementary Table S4) |

*Note:* Field 20003 was used to identify participants receiving lipid-lowering therapy (statins) based on ATC codes. PM2.5, particulate matter with aerodynamic diameter ≤2.5 μm; ICD, International Classification of Diseases.

***Supplementary Table S6. ICD-10 codes used for pancreatic disease outcome ascertainment***

| **Outcome** | **ICD-10 codes** | **Description** |
| --- | --- | --- |
| All pancreatic diseases | K85, K86, C25 | Composite of all pancreatic outcomes below |
| Acute pancreatitis | K85 | Acute pancreatitis |
| Chronic pancreatitis | K86.0, K86.1 | Chronic pancreatitis; other chronic pancreatitis |
| Pancreatic cysts | K86.2 | Cyst of pancreas |
| Pancreatic cancer | C25 | Malignant neoplasm of pancreas |
| Pancreas-related mortality | K85, K86, C25 | Death with pancreatic disease as underlying cause |

***Supplementary Materials 1 Methods of Bio/Phenotypic age and Bio/PhenoAgeAccel calculation***

Methodological details and analytical perspectives regarding these two biological age (BA) metrics are documented in prior publications (PMID: 34725754; PMID: 37120669). The estimation of BA was conducted with the "BioAge" R package (available at: <https://github.com/dayoonkwon/BioAge>).

The BioAge algorithm is grounded in the Klemera-Doubal method (KDM), employing a regression framework to model the relationship between biomarker profiles and chronological age (reference: 16318865). This approach involves calibrating an individual's physiological data against a reference distribution derived from the US National Health and Nutrition Examination Survey (NHANES III), which serves as the training set. To account for physiological variation between sexes, all model parameters were estimated independently for males and females. The BioAge formula is presented below:

In this formulation, *x* denotes the measured level of biomarker i for a given subject, while CA refers to chronological age. The coefficients k (intercept), q (slope), and s (root mean squared error) for each biomarker i are derived by regressing chronological age against the biomarker within the reference population. The scaling factor 𝑠BA corresponds to the square root of the variance in chronological age accounted for by the entire set of biomarkers in the reference sample.

The PhenoAge algorithm was developed through a comprehensive study of mortality risk trends (PMID: 30596641). Initially constructed using an elastic-net Gompertz regression model, it analyzed mortality outcomes in relation to 42 biomarkers from NHANES III. To distill this into a streamlined age estimator, the model underwent feature selection: the lambda parameter that minimized mean squared error was selected, resulting in the identification of nine key clinical markers. The PhenoAge equation is provided below:

As a measure of the divergence between biological age (BA) and chronological age, "age acceleration" was derived for both BioAge and PhenoAge. This was calculated as the regression residuals obtained from linearly modeling each BA metric against chronological age. Elevated values of BioAgeAccel and PhenoAgeAccel signify a state of accelerated biological aging. To reduce the influence of outliers, data points beyond ±5 standard deviations from the mean were removed from subsequent analysis.

***Supplementary Figure S1 Associations and Dose-Response Relationships of KDMAgeAccel with Pancreatic Disease Risk***

Panels A–F depict cumulative risk curves for overall pancreas diseases (A), acute pancreatitis (B), chronic pancreatitis (C), pancreatic cysts (D), pancreatic cancer (E), and pancreas-related mortality (F) across KDMAgeAccel groups. Higher KDMAgeAccel was associated with progressively increased cumulative risk. Panels G–L show Cox proportional hazards models with restricted cubic splines for KDMAgeAccel (treated as a continuous exposure), presenting overall and non‑linearity P values for overall pancreas diseases (G), acute pancreatitis (H), chronic pancreatitis (I), pancreatic cysts (J), pancreatic cancer (K), and pancreas-related mortality (L). A significant non‑linear association was observed only for chronic pancreatitis. Shaded areas indicate 95% confidence intervals (CI). All models were adjusted for age, sex, ethnicity, smoking status, alcohol intake frequency, physical activity, body mass index, sleep duration, educational attainment, Townsend deprivation index, PM₂.₅ exposure, diabetes mellitus, Cholelithiasis, and lipid‑lowering therapy use.

***Supplementary Figure S2 Association of Circulating Lipid Biomarkers with PhenoAgeAccel and Pancreatic Diseases***

(A) Cohen's d values for circulating lipid biomarkers between PhenoAgeAccel groups. Variables include total cholesterol, fatty acid unsaturation, VLDL and LDL subclasses, and lipid component ratios within lipoprotein particles. (B–D) Multivariable-adjusted hazard ratios (per 1-SD increment) for chronic pancreatitis (B), acute pancreatitis (C), and pancreatic cancer (D). All Cox regression models were adjusted for the same covariates as the primary analyses.
